# Identification of a potential gene target for osteoarthritis based on bioinformatics analyses

**DOI:** 10.1186/s13018-020-01756-w

**Published:** 2020-06-22

**Authors:** Zhi-xi Duan, Yu-sheng Li, Chao Tu, Peng Xie, Yi-han Li, Lin Qi, Zhi-hong Li

**Affiliations:** 1 Department of Orthopedics, The Second Xiangya Hospital, Central South University, 139 Renmin Road, Changsha, 410011 Hunan China; 2 Department of Orthopedics, Xiangya Hospital, Central South University, No.87 Xiangya Road, Changsha, 410008 China

**Keywords:** Osteoarthritis, PTHLH, GEO database, Differentially expressed genes, Protein–protein interaction network

## Abstract

**Background:**

Osteoarthritis (OA) is the most common chronic joint disease worldwide. It is characterized by pain and limited mobility in the affected joints and may even cause disability. Effective clinical options for its prevention and treatment are still unavailable. This study aimed to identify differences in gene signatures between tissue samples from OA and normal knee joints and to explore potential gene targets for OA.

**Methods:**

Five gene datasets, namely GSE55457, GSE55235, GSE12021, GSE10575, and GSE1919, were selected from the Gene Expression Omnibus (GEO) database. Differentially expressed genes (DEGs) were identified using the R programming software. The functions of these DEGs were analyzed, and a protein–protein interaction (PPI) network was constructed. Subsequently, the most relevant biomarker genes were screened using a receiver operating characteristic (ROC) curve analysis. Finally, the expression of the protein encoded by the core gene PTHLH was evaluated in clinical samples.

**Results:**

Eleven upregulated and 9 downregulated DEGs were shared between the five gene expression datasets. Based on the PPI network and the ROC curves of upregulated genes, PTHLH was identified as the most relevant gene for OA and was selected for further validation. Immunohistochemistry confirmed significantly higher PTHLH expression in OA tissues than in normal tissues. Moreover, similar PTHLH levels were detected in the plasma and knee synovial fluid of OA patients.

**Conclusion:**

The bioinformatics analysis and preliminary experimental verification performed in this study identified PTHLH as a potential target for the treatment of OA.

## Introduction

Osteoarthritis (OA) is the most prevalent chronic joint disease affecting adults [1–3]. It ultimately causes chronic pain, restricted joint mobility, and disability [4]. The most common risk factors for OA include age, sex, prior joint injury, obesity, genetic predisposition, and mechanical factors, including malalignment and an abnormal joint shape [5–8]. The pathogenesis of OA remains unclear, and no effective method is yet available to prevent its progression [9]. Therefore, considerable research attention has been directed toward the identification of effective biomarkers and potential therapeutic targets for OA.

An increased understanding of OA has revealed it to be a total joint disease, with characteristic features such as a narrowing of the joint space, articular cartilage degradation, osteophyte formation, synovial hyperplasia, and subchondral sclerosis [10]. However, the degeneration and loss of the cartilaginous extracellular matrix (ECM) and a reduction in the chondrocyte population remain the most important characteristic changes [11]. Previous research has shown that chondrocytes are the sole type of cells present in the cartilage, where they are responsible for both the synthesis and turnover of the ECM [12]. Therefore, an understanding of the genes relevant to maintaining chondrocyte health is very important for the prevention and treatment of OA.

High-throughput sequencing technology can provide information about the expression patterns of thousands of genes in the human genome simultaneously. In recent years, the rapid development of this technology has yielded a large amount of clinical sample data. These rich datasets have enabled researchers to better understand the molecular mechanism of OA, identify disease-related gene changes, and explore potential therapeutic targets [13, 14]. However, due to heterogeneity among samples or sequencing platforms, mRNA expression data may not be consistent with different gene maps. Consequently, sequencing dataset analyses that have focused on OA have not yielded reliable results. A comprehensive bioinformatics approach would likely address the above-described shortcomings of previous work and enable the identification of the key genes involved in OA.

In this study, we selected five mRNA datasets (GSE55457, GSE55235, GSE12021, GSE10575, and GSE1919) closely related to OA from the GEO database. First, we used comprehensive bioinformatics analyses to identify DEGs between OA and normal samples. Next, we subjected these DEGs to functional enrichment and pathway analyses and constructed a PPI network, which was screened for key genes. An OA-specific biological function and pathway analysis may further our understanding of the mechanism of OA development at the molecular level. This knowledge could further promote our understanding of OA pathogenesis, in turn improving diagnosis, prognosis, and drug target identification.

## Materials and methods

### Data sources

The following gene expression profile datasets were downloaded from the GEO database: GSE55457, GSE55235, GSE12021, GSE10575, and GSE1919. The five GEO gene datasets contained 37 OA samples and 36 control samples (the corresponding information is shown in Table 1); all rheumatoid arthritis (RA) samples were excluded. The profiles of GSE55457, GSE55235, and GSE12021 were based on the GPL96 [HG-U133A] Affymetrix Human Genome U133A Array. The profile of GSE10575 was based on the GPL570 [HG-U133_Plus_2] Affymetrix Human Genome U133 Plus 2.0 Array. The profile of GSE1919 was based on the GPL91 [HG_U95A] Affymetrix Human Genome U95A Array.

**Table 1:** Sample statistics of the five GEO gene datasets

Dataset ID	OA	Control	Total
GSE55457	10	10	20
GSE55235	10	10	20
GSE12021	10	9	19
GSE1919	5	5	10
GSE10575	2	2	4

### Identification of DEGs

The limma package for the R platform was used to screen for DEGs between OA and normal knee joints, including synovial tissue samples (OA, 35; normal, 34) and cell samples (healthy chondrocytes, 2; OA osteocytes, 2). A *P* value < 0.05 and |log2FC| > 1 were used as the screening criteria for both upregulated and downregulated DEGs. Venn diagrams of genes shared between and unique to the five datasets were drawn using the R package. We also standardized the log2FC values of the genes shared between the five datasets and used the R package pheatmap to produce a cluster analysis heatmap of these shared genes.

### GO enrichment and KEGG pathway analyses

Next, Gene Ontology (GO, http://www.geneontology.org/) and Kyoto Encyclopedia of Genes and Genomes (KEGG, http://www.genome.jp/kegg/pathway.html) enrichment analyses were performed using the R package clusterProfiler to detect the functions and pathways affected by the DEGs. The R package ggplot2 was used to plot the results for which KEGG annotation entries existed in at least four datasets, and for which GO subclass annotation entries existed in at least three datasets according to the annotation statistics contained in each dataset.

### PPI network construction

To construct the PPI network of shared DEGs, we used the Search Tool for the Retrieval of Interacting Genes (STRING), an online database. Using the Cytoscape3.6.0 software, the gene with the highest degree of connectivity to the surrounding genes was selected based on the PPI network.

### Collection of clinical samples and culture of chondrocytes

Twelve samples of knee cartilage were obtained from human patients who had been diagnosed with primary knee OA (grades III–IV) and underwent total knee arthroplasty. Six normal knee cartilage specimens were collected from patients with osteosarcoma who underwent segmental resection and artificial tumor knee prosthesis replacement or trauma amputation surgery. The clinical sample information is shown in Table 2. The study was conducted in accordance with the Declaration of Helsinki (2000 revision), and the protocol was approved by the Ethics Committee of the Second Xiangya Hospital, Central South University in Changsha, China (No. 2019106). The primary culture of human chondrocytes was the same as reported previously [15]. Cultured chondrocytes from no later than the first passage was used for the experiments.

**Table 2:** Patient information of knee cartilage and chondrocytes samples

Characteristic	OA	Control (normal)	*P* value
N	12	6	
Male	4	4	
Female	8	2	
Age (years)	66.83 ± 6.38, (range 57–75)	21.33 ± 10.09, (range 8–39)	< 0.001
BMI (kg/m^2^)	24.57 ± 2.34, (range 22.03–29.97)	20.05 ± 2.13, (range 17.97–2.13)	< 0.05

### Immunohistochemistry (IHC) and immunocytochemistry (ICC)

Six OA samples were randomly selected from 12 OA cartilage samples and verified with 6 normal cartilage samples. Cartilage tissues were fixed in 4% paraformaldehyde for 48 h and then decalcified in 10% ethylenediaminetetraacetic acid for approximately 4 weeks. Each tissue was embedded in paraffin and sectioned at a thickness of 6 μm. The experimental process was performed as previously described [16]. Additionally, 6 OA chondrocytes samples were randomly selected from 12 OA chondrocytes samples and verified with 6 normal chondrocytes samples. Human primary chondrocytes were seeded into chamber slides and then fixed with 4% paraformaldehyde for 10 min, permeabilized with 0.1% Triton X-100 in phosphate-buffered saline (PBS) for 15 min, and treated with 1% bovine serum albumin–PBS for 30 min. All fixed tissues and cells were incubated with a rabbit anti-PTHLH antibody (Santa Cruz Biotechnology, USA) for 2 h, followed by a peroxidase-conjugated anti-goat IgG secondary antibody for 30 min. The nuclei were counterstained with hematoxylin. All procedures were performed at room temperature.

### Enzyme-linked immunosorbent assay (ELISA)

The blood samples of 20 normal healthy people came from the Health Management Center of the Second Xiangya Hospital, and their physical examination results showed that they were healthy. The blood and synovial fluid samples of 23 OA patients came from the Department of Orthopedics of the Second Xiangya Hospital. Their physical examination results showed that there was no parathyroid-related disease. Blood and synovial fluid samples were collected, centrifuged, and stored immediately at − 80 °C until analysis. The clinical sample information is shown in Table 3. The PTHLH concentrations in the plasma and synovial fluid were determined using a commercial ELISA (USCN Life Science & Technology Co Ltd., Wuhan, China) according to the manufacturer’s instructions.

**Table 3:** Patient information of blood samples and synovial fluid samples

Characteristic	OA	Normal (healthy)	*P* value
N	23	20	
Male	7	10	
Female	16	10	
Age (years)	69.78 ± 6.80, (range 57–85)	30 ± 4.41, (range 22–38)	< 0.001
BMI (kg/m^2^)	24.45 ± 2.80, (range 16.65–29.97)	20.96 ± 1.95, (range 17.72–24.62)	< 0.001

### Statistical analysis

R version 3.5.3 was used for the statistical analyses of the GEO datasets. In addition, the GraphPad Prism Software, version 7.0 was used for the statistical analysis of experimental data. The results are expressed as means ± standard deviations (SDs). A receiver operating characteristic (ROC) curve analysis was performed to identify possibly optimal models and discard suboptimal ones independently of (and prior to specifying) the cost context or the class distribution. Unpaired *t* test was used to analyze the statistical differences in age and BMI between OA group and control group. In addition, we used D’Agostino-Pearson, Shapiro-Wilk, and Kolmogorov–Smirnov normality test to test the normal distribution of data. Statistical analysis of immunohistochemical data showed that the data are normal distribution, although the sample size is relatively small. However, the statistical analysis of ELISA data showed that the data are not normal distribution. Therefore, we choose Mann-Whitney *U* test for statistical analysis. *P* < 0.05 was considered to indicate a significant difference between the groups.

## Results

### Identification of 20 DEGs shared between five GEO profiles

**Fig. 1:**
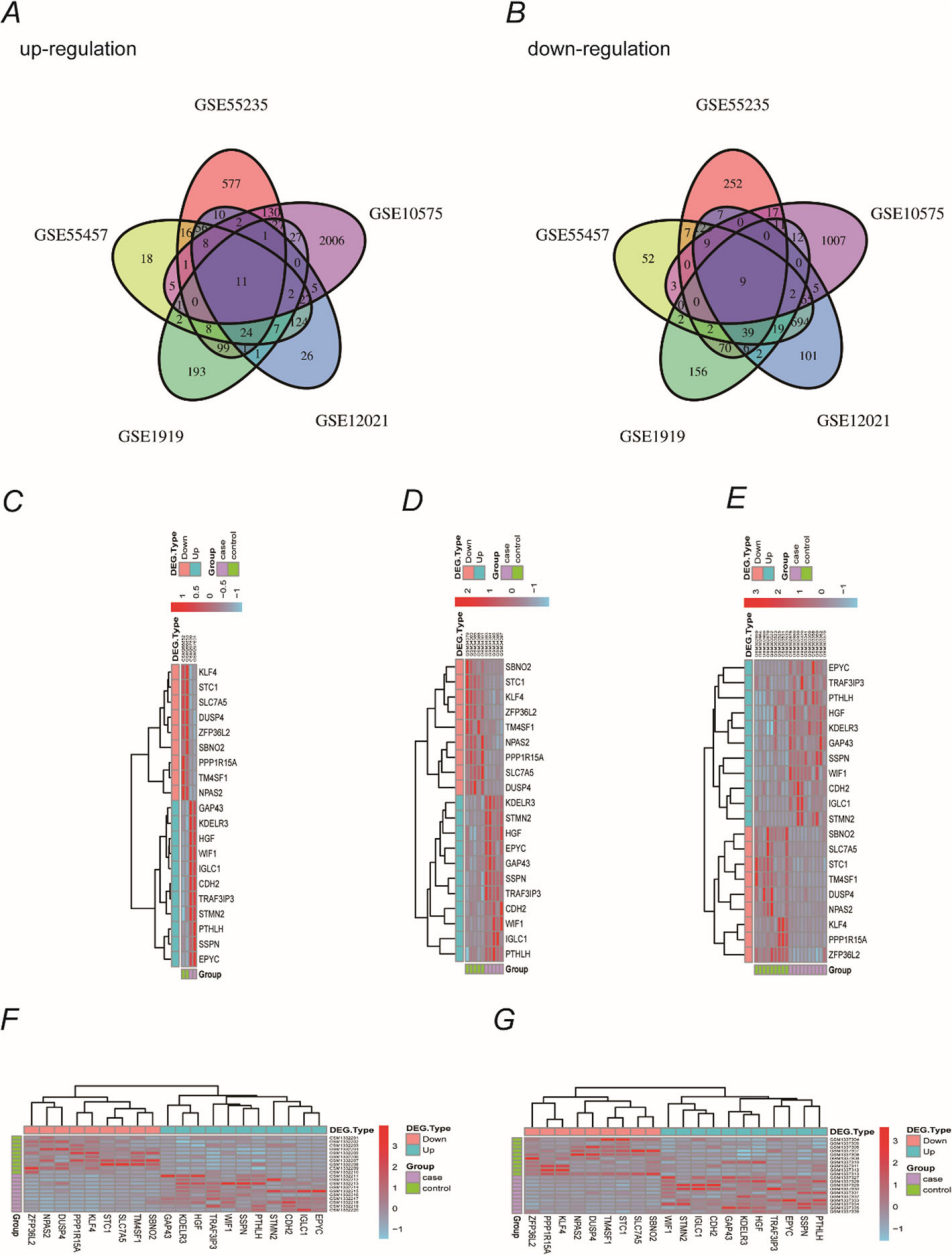
DEGs screened from five GEO datasets. **a** Eleven upregulated DEGs and **b** nine downregulated were shared between the five GEO datasets. **c**–**g** Cluster analysis heatmaps of the 20 shared DEGs in each dataset

We first analyzed the DEGs in the five GEO gene datasets and identified 1334, 1521, 1384, 3393, and 732 DEGs, including 309, 970, 304, 2253, and 403 upregulated genes and 1025, 551, 1080, 1140, and 329 downregulated genes, in GSE55457, GSE55235, GSE12021, GSE10575, and GSE1919, respectively. Twenty DEGs, including 11 upregulated and 9 downregulated genes, were shared between all five datasets, as shown by Venn diagrams in Fig. 1 a and b. In addition, we performed a cluster analysis of these 20 common DEGs in each dataset (Fig. 1c–g). As shown in the figure, downregulated and upregulated genes are represented in pink and blue, respectively. The OA and normal groups are depicted in purple and green, respectively, while red and blue coloring indicates high and low expression levels, respectively.

### GO functional enrichment analysis

**Fig. 2:**
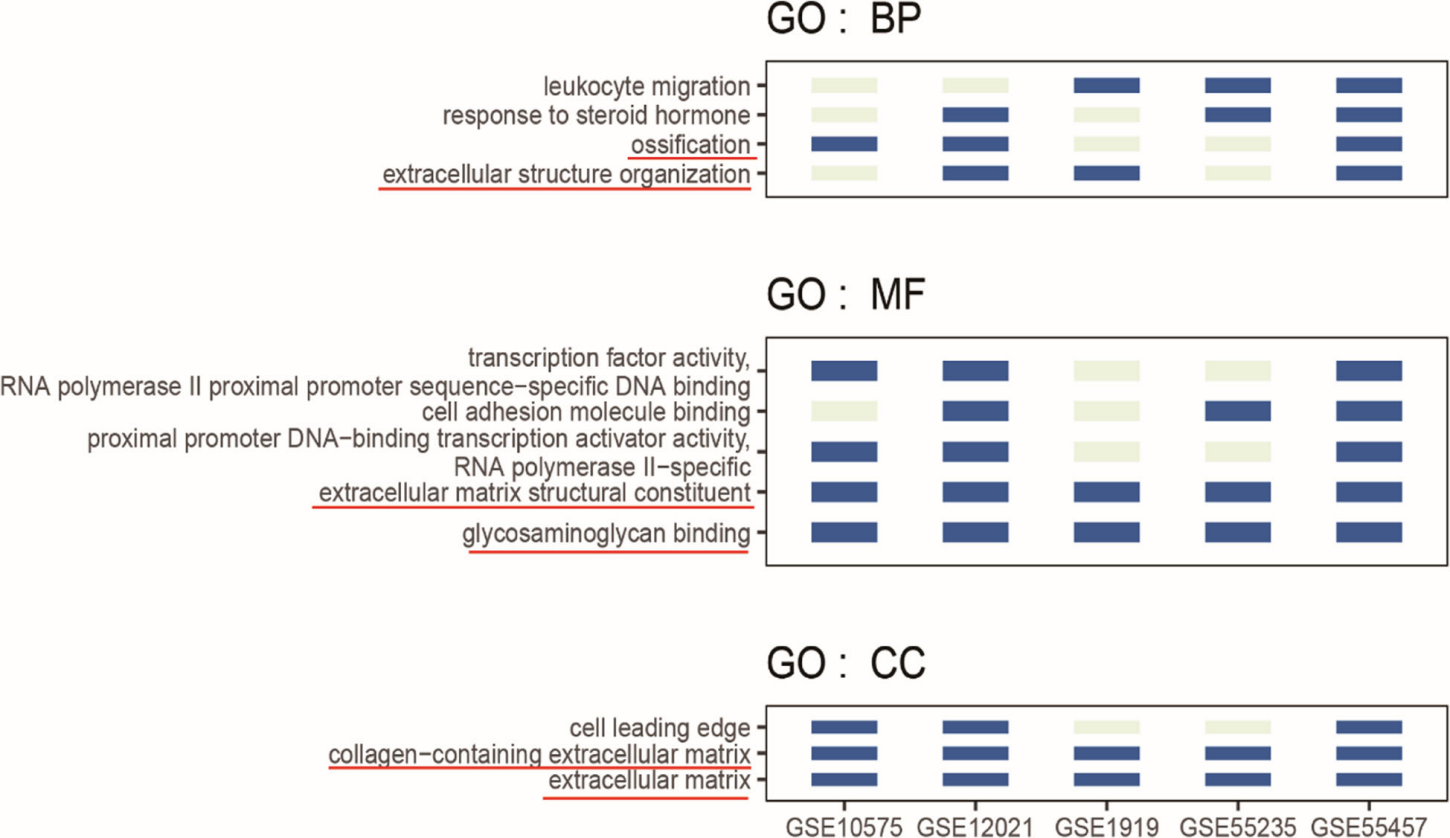
GO enrichment analyses of DEGs. The image indicates GO subclass annotations existing in at least three datasets (dark blue columns of bars indicate). The shared genes classified into the molecular function (MF), biological processes (BP), and cellular component (CC) categories are summarized

Next, a GO functional enrichment analysis was performed using the R package clusterProfiler to identify the functions of the DEGs, which were classified into the groups of biological processes (BP), cellular component (CC), and molecular function (MF) (Fig. 2). Notably, the common genes were significantly associated with several GO terms. Particularly, the most strongly enriched BP terms included “leukocyte migration,” “response to steroid hormone,” “ossification,” and “extracellular structure organization.” The most strongly enriched MF terms were “extracellular matrix structural constituent” and “glycosaminoglycan binding.” The most common CC terms were “cell leading edge,” “collagen-containing extracellular matrix,” and “extracellular matrix.” These functions are known to be closely related to OA.

### KEGG pathway enrichment analysis

**Fig. 3:**
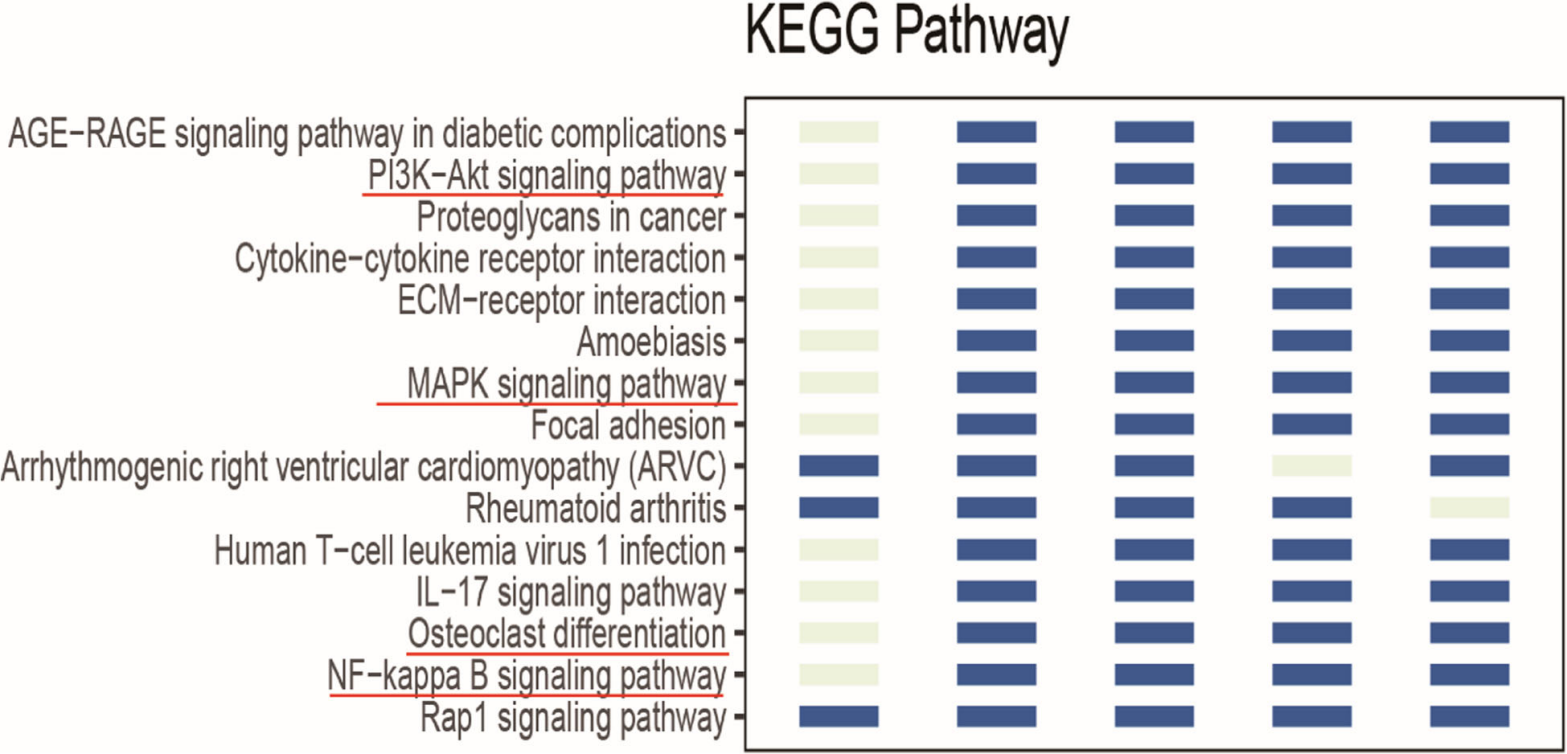
Results of the KEGG pathway enrichment analyses of DEGs. KEGG annotation entries existed in at least four datasets (dark blue columns of bars indicate) according to the annotation statistics

A KEGG pathway enrichment analysis revealed that the shared genes were mainly enriched in OA-related signaling pathways, including the PI3K-AKT, MAPK, and NF-kappa B signaling pathways, as well as in osteoclast differentiation [17–19] (Fig. 3).

### PPI network analysis

**Fig. 4:**
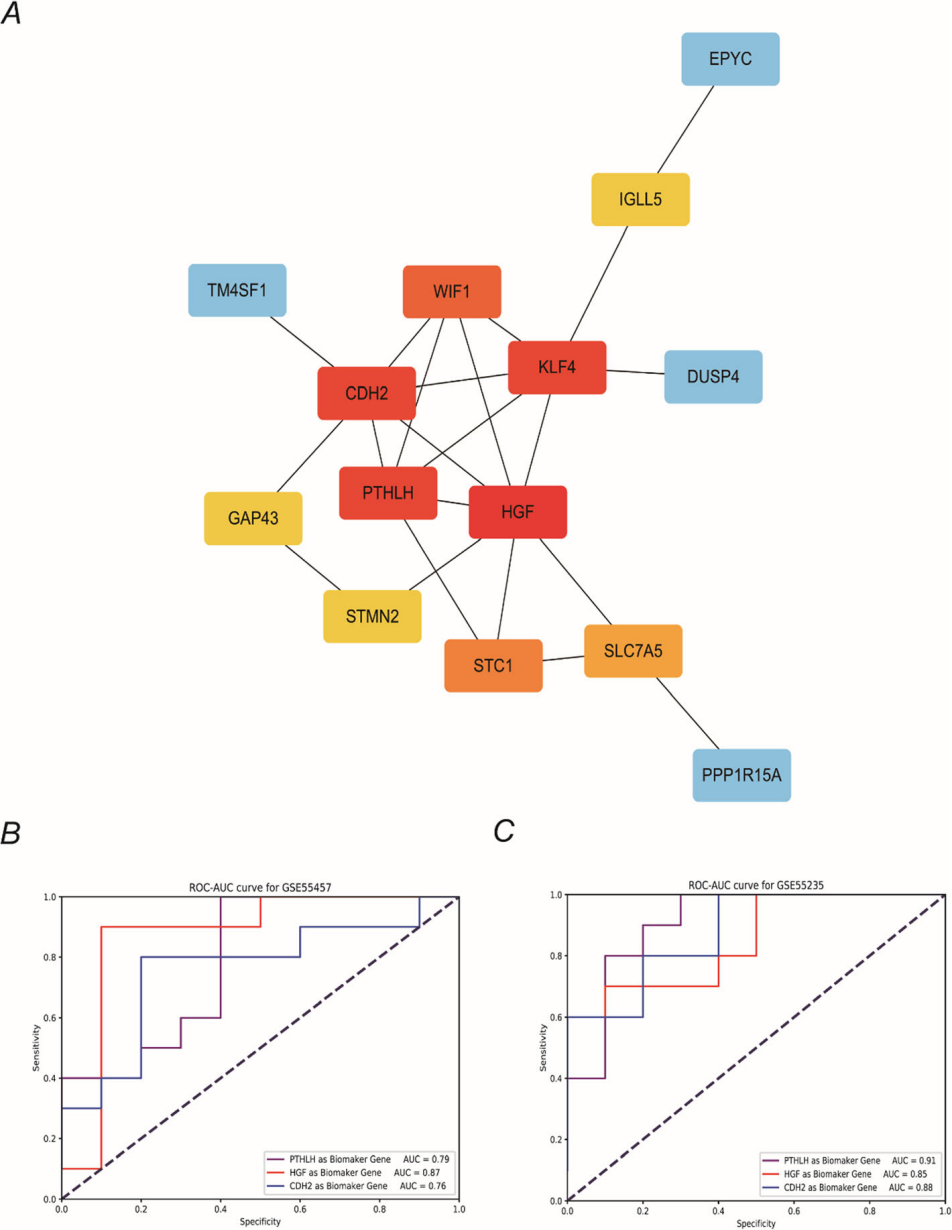
Construction of a PPI network using the DEGs. **a** Schematic of the PPI network constructed from the DEGs. The scores are indicated by colors, with the top 5 DEGs indicated in red. The ROC-AUCs for **b** GSE55457 and **c** GSE 55235

To reveal the interactive relationships between proteins encoded by the DEGs, we constructed a PPI network of the 20 shared DEGs using STRING and Cytoscape software (Fig. 4a). Here, the top 10 hub genes were *HGF*, *PTHLH*, *CDH2*, *KLF4*, *WIF1*, *STC1*, *SLC7A5*, *IGLL5*, *STMN2*, and *GAP43*. Four of the top five genes were found to be upregulated (*HGF*, *PTHLH*, *CDH2*, *WIF1*) and were thus selected for further analysis. Subsequently, we identified the upregulated genes (*HGF*, *PTHLH*, and *CDH2*) with higher scores in the sub-network analysis and selected the best potential target gene using a ROC curve. As shown in Fig. 4 b and c, in the GSE 55457 data set, HGF (AUC = 0.87) has the largest AUC area. However, there are many studies on the relationship between HGF and osteoarthritis, and the results are controversial or even contradictory [20–22]. Therefore, we finally decided to choose PTHLH (AUC = 0.91) in the GSE55235. These findings suggest that PTHLH is the most relevant biomarker for OA.

### IHC and ICC validation of the gain of PTHLH expression

**Fig. 5:**
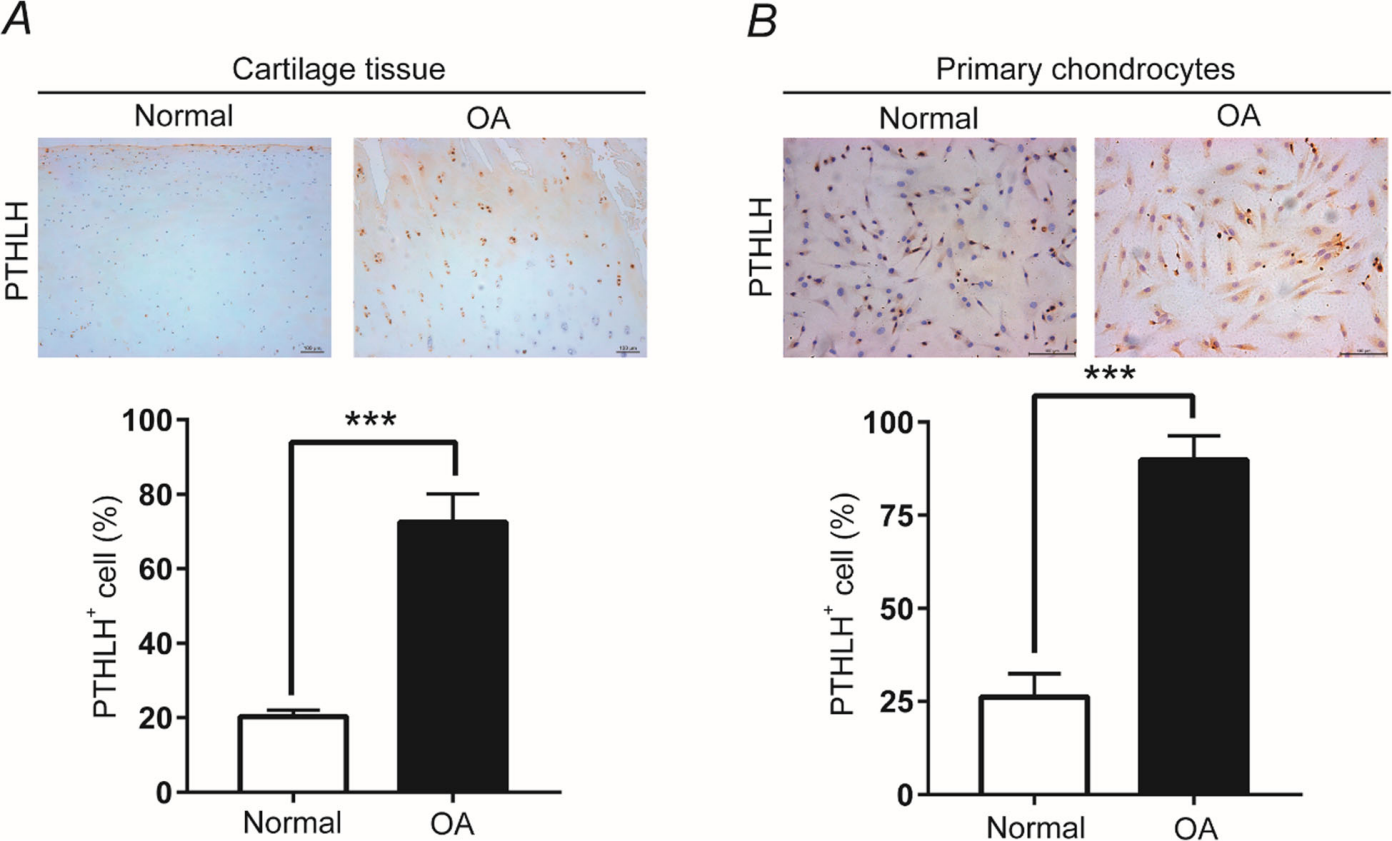
Increased PTHLH expression in **b** OA cartilage and **b** OA chondrocytes. The numbers of PTHLH (+) cells in cartilage tissues and primary chondrocytes subjected to IHC are presented as means ± SDs in the lower panel. Student’s *t* test. **a** ***P < 0.001, *n* = 6 (scale bars = 100 μm; magnification: × 10). **b** **P < 0.01, *n* = 6 (scale bars = 100 μm; magnification: × 20)

To confirm the high expression of PTHLH in relation with OA, we subjected samples of normal and OA cartilage to IHC. Notably, the PTHLH expression was significantly higher in OA cartilage than in normal cartilage (*P* < 0.001; Fig. 5a). We also assessed PTHLH expression in normal and OA primary chondrocytes and found higher expression in the latter (*P* < 0.01; Fig. 5b).

### PTHLH concentrations in the plasma and synovial fluid

**Fig. 6:**
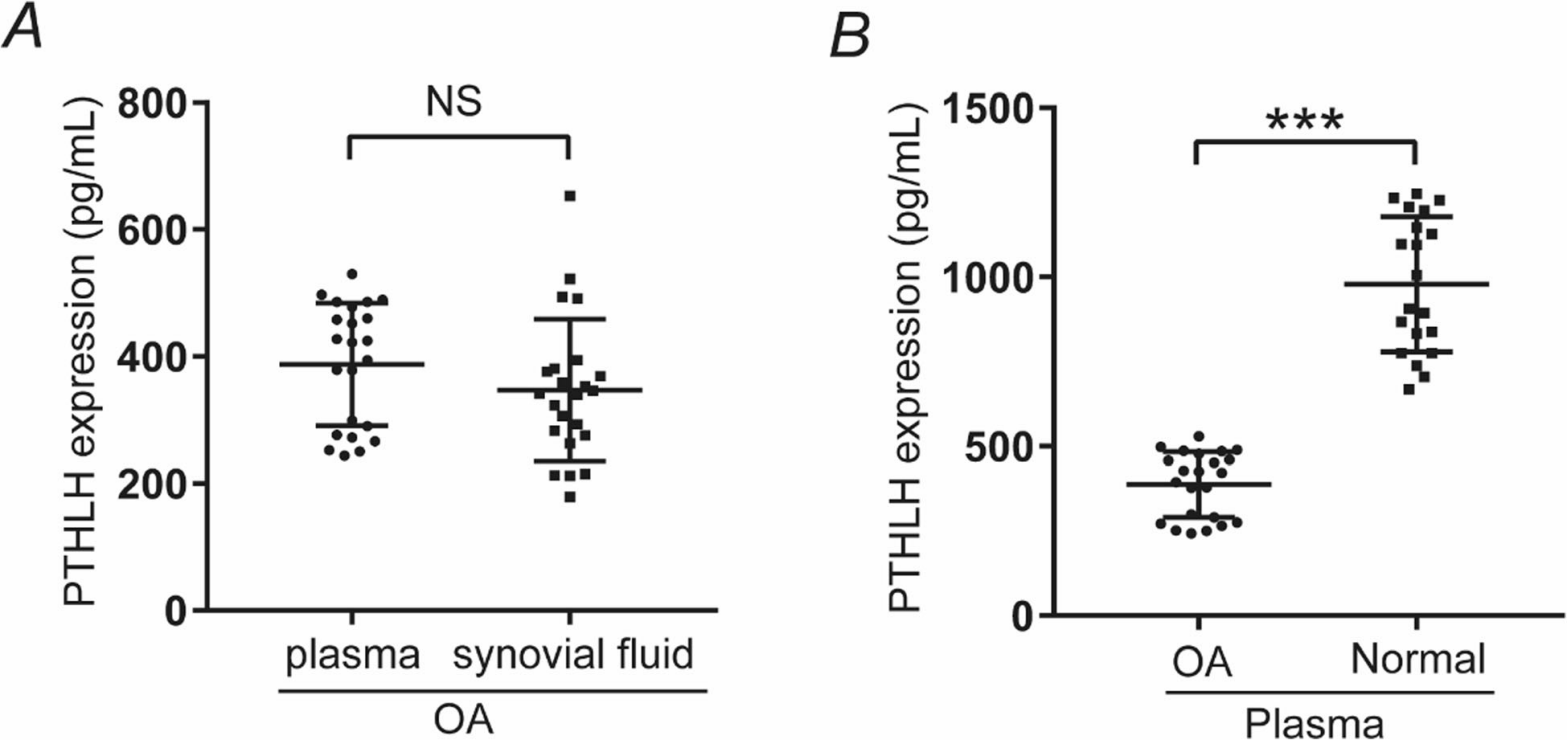
PTHLH levels in the plasma and synovial fluid. **a** PTHLH levels in the plasma and synovial fluid samples of OA patients, Mann-Whitney *U* test, *P* > 0.05. **b** PTHLH levels in the plasma samples of healthy and OA patients, Mann-Whitney *U* test, ***P < 0.001

The healthy human knee cavity contains only a small amount of synovial fluid, which is almost impossible to obtain. In contrast, OA patients exhibit a significantly increased amount of synovial fluid. Thus, we used ELISA to evaluate the PTHLH levels in the synovial fluid and plasma of OA patients. Notably, no significant differences were observed in the PTHLH levels between these sample types (*P* > 0.05; Fig. 6a). We also measured the PTHLH levels in the plasma samples of healthy people and found significantly higher levels in them compared with those in the plasma samples of OA patients (*P* < 0.001; Fig. 6b).

## Discussion

OA is one of the leading causes of disability worldwide and thus places heavy burdens on the healthcare systems and global economy [23, 24]. Although the early diagnosis and treatment of OA are important, the mechanisms underlying OA pathogenesis remain unclear. It is essential to understand OA pathogenesis as this knowledge will facilitate diagnosis, prognosis, and drug target discovery [25–27]. High-throughput sequencing and microarray technology have been widely used to predict potential diagnostic and therapeutic targets for OA. In this study, we identified 20 genes that were differentially expressed between OA and normal samples and common to all five selected GEO mRNA expression datasets. Through GO and KEGG enrichment analyses, we determined that the processes affected by these genes were consistent with the findings of previous studies in which these pathways were identified as contributors to the pathological process of OA [28–30].

We also constructed PPI networks containing these DEGs. Among the top 10 hub genes, namely *HGF*, *PTHLH*, *CDH2*, *KLF4*, *WIF1*, *STC1*, *SLC7A5*, *IGLL5*, *STMN2*, and *GAP43*, previous studies identified HGF as a multifunctional contributor to OA. On the one hand, HGF can promote synovial and vascular proliferation, leading to inflammation and osteophyte formation [20, 22, 31]; on the other hand, it supports bone and cartilage repair [21]. Other reports have described CDH2 polymorphisms and KLF4 as potentially associated with the development of OA [32, 33]. PTHLH has been identified primarily as a factor that promotes chondrocyte proliferation and inhibits chondrocyte hypertrophy and terminal differentiation [34, 35]. Few reports have described correlations of the remaining hub genes with OA.

**Fig. 7:**
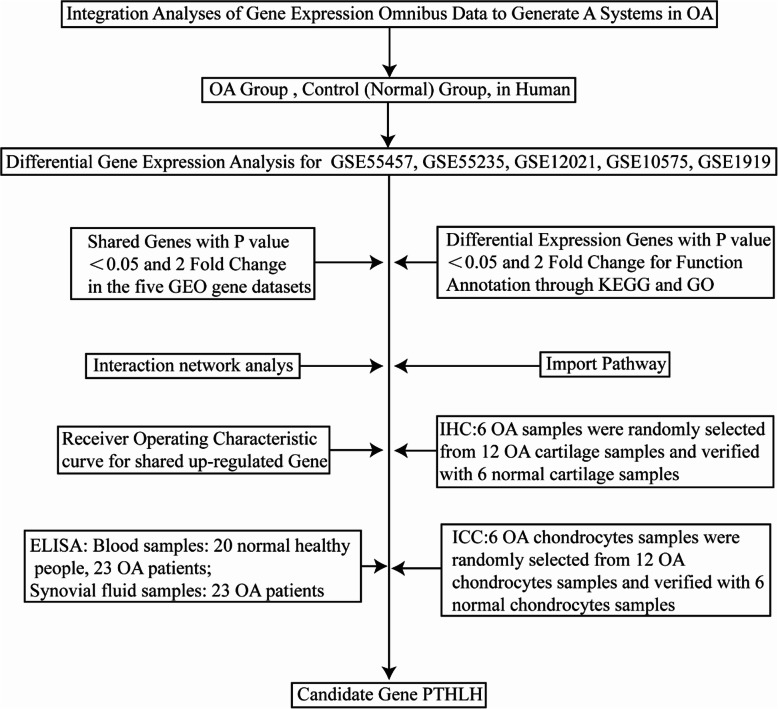
Flowchart of the study

Our bioinformatics analysis results and existing literature evidence led us to speculate that PTHLH is a diagnostic and predictive biomarker of OA. Our IHC analysis to validate the aberrant gain-of-expression of PTHLH in OA cartilage tissue confirmed the significantly increased PTHLH levels in OA cartilage tissues and chondrocytes relative to those in normal control samples. However, an ELISA-based experiment revealed a significantly higher PTHLH level in the plasma of healthy people than in the plasma of OA patients. We evaluated why the PTHLH level is higher in OA knee cartilage or chondrocytes than in their normal counterparts and why the plasma PTHLH level is lower in OA patients than in normal people and identified the following possible reasons. First, under normal circumstances, PTHLH (the protein encoded by *PTHLH*, also known as PTHrP) plays predominantly paracrine and/or autocrine roles [36]. PTHLH exerts paracrine actions that affect physiological homeostasis in many tissues and processes, including hair follicles, cartilage, vascular smooth muscle, bone, pancreas, mammary gland development, and tooth eruption [35]. The secretion of PTHLH in some tissues and organs, such as the vascular smooth muscle, was shown to decrease with aging [37], and most patients with OA tend to be older [5]. Second, chondrocytes and synovial tissues can produce PTLHL directly [38, 39]. Therefore, in the pathological state of OA, chondrocytes and synovial tissues may secrete more PTHLH to maintain their own cell activity (Fig. 7).

Our study had some limitations. Firstly, there were statistical differences in age and BMI between the OA group and the control group (Table 2 and Table 3). At present, there are few studies on BMI and PTHLH levels. We are not sure whether BMI will affect PTHLH level. We emphasize that the biggest weakness of the study is that we do not know if OA itself causes the differences or the differences of age and BMI in the groups. Therefore, we designed a study that can obviate this issue. In future experiments, we will collect clinical samples in groups according to different age stages and different BMI intervals, and expand the sample size as much as possible. Secondly, although our analysis included five public datasets, the sample size was relatively small. In addition, the sample size used to verify the PTHLH expression data was also relatively small. Further experimental studies with larger sample sizes are needed to confirm our results.

## Conclusion

In summary, we used bioinformatics analysis to identify 20 DEGs between normal and OA cartilage tissues and identified PTHLH as the main upregulated gene in the resulting PPI network. Our findings indicate that PTHLH may serve as a potential therapeutic target.

## Data Availability

The datasets used and/or analyzed during the current study are available from the corresponding author on reasonable request.

## Abbreviations

OA: Osteoarthritis
DEGs: Differentially expressed genes
PPI: Protein–protein interaction
ROC: Receiver operating characteristic
ECM: Extracellular matrix
GO: Gene Ontology
KEGG: Kyoto Encyclopedia of Genes and Genomes
STRING: Search Tool for the Retrieval of Interacting Genes
BP: Biological processes
CC: Cellular component
MF: Molecular function
